# Capture-Based Next-Generation Sequencing Improves the Identification of Immunoglobulin/T-Cell Receptor Clonal Markers and Gene Mutations in Adult Acute Lymphoblastic Leukemia Patients Lacking Molecular Probes

**DOI:** 10.3390/cancers12061505

**Published:** 2020-06-09

**Authors:** Roberta Cavagna, Marie L. Guinea Montalvo, Manuela Tosi, Michela Paris, Chiara Pavoni, Tamara Intermesoli, Renato Bassan, Andrea Mosca, Alessandro Rambaldi, Orietta Spinelli

**Affiliations:** 1 Hematology and Bone Marrow Transplant Unit, Azienda Socio-Sanitaria Territoriale (ASST) Papa Giovanni XXIII, 24127 Bergamo, Italy; roberta.cavagna@unimi.it (R.C.); marielorena@gmail.com (M.L.G.M.); mtosi@asst-pg23.it (M.T.); michela.paris90@gmail.com (M.P.); cpavoni@asst-pg23.it (C.P.); tintermesoli@asst-pg23.it (T.I.); ospinelli@asst-pg23.it (O.S.); 2 Department of Pathophysiology and Transplantation, Università degli Studi di Milano, 20122 Milano, Italy; andrea.mosca@unimi.it; 3 Hematology Unit, dell’Angelo Hospital and SS. Giovanni and Paolo Hospital, 30174 Venezia Mestre, Italy; renato.bassan@aulss3.veneto.it; 4 Department of Oncology and Hematology, Università degli Studi di Milano, 20122 Milano, Italy

**Keywords:** acute lymphoblastic leukemia, next-generation sequencing, minimal residual disease

## Abstract

The monitoring of minimal residual disease (MRD) in Philadelphia-negative acute lymphoblastic leukemia (ALL) requires the identification at diagnosis of immunoglobulin/T-cell receptor (Ig/TCR) rearrangements as clonality markers. Aiming to simplify and possibly improve the patients’ initial screening, we designed a capture-based next-generation sequencing (NGS) panel combining the Ig/TCR rearrangement detection with the profiling of relevant leukemia-related genes. The validation of the assay on well-characterized samples allowed us to identify all the known Ig/TCR rearrangements as well as additional clonalities, including rare rearrangements characterized by uncommon combinations of variable, diversity, and joining (V-D-J) gene segments, oligoclonal rearrangements, and low represented clones. Upon validation, the capture NGS approach allowed us to identify Ig/TCR clonal markers in 87% of a retrospective cohort (MRD-unknown within the Northern Italy Leukemia Group (NILG)-ALL 09/00 clinical trial) and in 83% of newly-diagnosed ALL cases in which conventional method failed, thus proving its prospective applicability. Finally, we identified gene variants in 94.7% of patients analyzed for mutational status with the same implemented capture assay. The prospective application of this technology could simplify clonality assessment and improve standard assay development for leukemia monitoring, as well as provide information about the mutational status of selected leukemia-related genes, potentially representing new prognostic elements, MRD markers, and targets for specific therapies.

## 1. Introduction

In the last decade, the monitoring of molecular minimal residual disease (MRD) has become the gold standard in routine clinical practice both in pediatric and adult acute lymphoblastic leukemia (ALL) to improve risk stratification and define therapeutic strategies [1,2,3,4,5]. Indeed, the evaluation of MRD during induction and maintenance chemotherapy, or after hematopoietic stem cell transplantation (HSCT), is nowadays considered the strongest predictive factor for ALL prognosis [6,7]. The availability of MRD data relies on a standardized, time-consuming, and intense procedure applying PCR amplification to detect the most frequent immunoglobulin/T-cell receptor (Ig/TCR) gene rearrangements and leukemic fusion transcripts at diagnosis [8,9,10,11]. However, in about 5-10% of cases, the use of this method fails to identify suitable markers for MRD evaluation [12], precluding patients’ allocation in the most adequate risk class which is crucial to define the most suitable therapy and to promptly prevent relapse. This failure can be attributed to the PCR technique limitation concerning both the design and resolution of the assay. As a matter of fact, the large number of possible combinations of variable, diversity, and joining (V-D-J) gene segments is faced by PCR with a restricted number of primer combinations that can be used to affordably assess clonal rearrangement identification due to diagnostic DNA availability, time to assay set-up, and costs. Therefore, a PCR-based panel designed for clonality assessment at disease presentation is ineffective in identifying rare rearrangements and in solving oligoclonality/low clonality issues.

New high-throughput technologies have become available and are being explored in order to overcome the limits of the conventional and standardized MRD assessment [13]. A next-generation sequencing (NGS) approach, similarly based on rearrangement amplification by PCR, has been described to identify clonal markers at diagnosis [14,15,16] and to monitor MRD in lymphoid malignancies [17,18,19,20,21]. All in all, NGS-based Ig/TCR marker screening greatly improved MRD monitoring, being less time consuming, and highlighted the polyclonal false-positive scenario. An alternative to amplicon-based NGS for Ig/TCR marker screening is a capture enrichment NGS procedure based on the hybridization of probes selecting all the known V, D, and J regions irrespective of their recombination status [22]. The capture method also allows for the inclusion of highly complex (i.e., TCRA) or preferentially amplified Ig/TCR regions, which can hinder multiplex PCR technique [14]. Therefore, this approach can potentially identify all the possible rearrangements also offering, by a single test and using the same amount of specimen, the possibility to include other probes recognizing leukemia-related DNA regions such as chromosomal translocation partners [22] or genes with prognostic significance in hematologic malignancies [23]. Recently, many molecular alterations have been suggested as possible candidates for predicting ALL outcome [24], or defining precise features such as Philadelphia chromosome-like leukemia [25].

In this context, we developed a custom capture-based NGS approach for Ig/TCR clonal marker identification at disease onset, also including the analysis of the mutational status of 25 genes relevant in diagnostic workup and prognosis of ALL [24]. We aimed to demonstrate its value in identifying common and uncommon Ig/TCR rearrangements as well as gene mutational status and its prospective applicability to better define risk class.

## 2. Results

### 2.1. Validation of the Capture-Based NGS Panel

We applied a newly designed capture-based NGS assay (panel v1, see Materials and Methods) to 10 previously characterized diagnostic samples. By comparing both Ig/TCR annotation and complementarity-determining region 3 (CDR3) sequence, we identified 51 rearrangements already found by standard PCR-based clonality assessment and Sanger sequencing. This novel approach also detected 24 additional clonal rearrangements involving the following loci: IGH (*n* = 4), IGK (*n* = 2), IGL (*n* = 1), TCRB (*n* = 4), TCRG (*n* = 2), and TCRA and TCRD (*n* = 11). These clonal rearrangements were mainly characterized by uncommon V-D-J combinations, low clonal representation, and oligoclonality that could not be solved by the conventional homo-heteroduplex clonality assessment (Table 1). 

The validation of these NGS-recognized rearrangements using standard methods required further efforts compared to routine procedures—11 uncommon V-D-J combinations were amplified by rearrangement-specific primers designed based on NGS data (*n* = 5 in TCRA, *n* = 1 in TCRD, and *n* = 5 in αδ (TCRA+D) loci) and sequenced by Sanger method; three low-represented clonalities (*n* = 1 IGH, *n* = 1 TCRB, and *n* = 1 IGL) that were missed by low sensitive regular PCR were finally revealed by standard method only after re-amplification of faint heteroduplex PCR products and Sanger sequencing; 10 oligoclonal rearrangements (*n* = 2 IGK, *n* = 3 IGH, *n* = 3 TCRB, and *n* = 2 TCRG) were amplified and recognized within the typical, overlapped Sanger electropherograms. Notably, the analysis of NGS data derived from human umbilical vein endothelial cells (HUVECs) and mesenchymal cord blood cells samples did not identify any rearrangement in Ig/TCR genes, testifying that no false rearrangements were produced during library preparation, sequence generation, or data analysis.

### 2.2. Retrospective Application of the Capture-Based NGS Panel to MRD-Unknown ALL Patients

The capture-based NGS assay (panel v1) was then applied to a retrospective group of 23 ALL patients (13 B-lineage ALL and 10 T-lineage ALL) enrolled into the Northern Italy Leukemia Group (NILG)-ALL 09/00 clinical trial for whom it had been impossible to isolate clonal rearrangements or to obtain a sensitive patient-specific molecular probe (MRD-unknown). These patients could not have benefited from an MRD driven treatment and were treated according to the clinical risk score. In this cohort, the capture-based NGS allowed for the identification of at least 1 rearrangement in 20 out of 23 patients (87%), recognizing overall 92 clonal rearrangements (IGH (*n* = 30), IGK (*n* = 6), TCRB (*n* = 6), TCRG (*n* = 32), and TCRA and TCRD (*n* = 18)) detailed in Table 2.

In all 13 adult B-lineage ALL cases, we recognized by NGS at least one D-J or V-D-J clonal rearrangement, identifying a total of 72 rearrangements. On the contrary, our approach identified D-J or V-D-J clonal rearrangements in 7 out of 10 adult T-lineage ALL cases. Among the complete cohort of MRD unknown patients, we found 21 out of 92 rearrangements at level <5% that were not subjected to validation due to the 5% detection limit of standard procedure. Conversely, 69 rearrangements defined by NGS with a level ≥5% underwent the validation process by the conventional method. Among these 69 cases, 53 were successfully amplified and Sanger sequenced using implemented standard PCR methods based on the updated EuroMRD guidelines or specific PCR appropriately designed for uncommon rearrangements based on NGS sequences. For 16 out of 69 rearrangements, we could not confirm the NGS sequences by conventional Sanger sequencing because it does not allow for the discrimination of clones sharing the same V-J genes but with different junctional regions. Confirmation was not possible for two rearrangements identified in one sample due to diagnostic DNA exhaustion.

### 2.3. Prospective Application of the Capture-Based NGS Panel and Patient-Specific Probe Design

The prospective application of our capture-based NGS panel allowed for the identification of at least one rearrangement in 10 out of 12 patients (83%), with a total of 40 clonal rearrangements identified, located in the following loci: IGH (*n* = 4), IGK (*n* = 6), IGL (*n* = 3), TCRB (*n* = 5), TCRG (*n* = 14), and TCRA and TCRD (*n* = 8) (Table 3).

In all the three adult B-lineage ALL studied cases, we recognized D-J or V-D-J clonal rearrangements by NGS capture approach, identifying overall 18 rearrangements, including seven clonal rearrangements already isolated by conventional method, but not adequate to patient-specific probe generation. Among the nine remaining adult T-lineage cases, we identified D-J or V-D-J clonal rearrangements in seven patients. Overall, the newly-identified rearrangements were again mainly characterized by an uncommon V-D-J combination. In addition, an IGK rearrangement, routinely tested exclusively in B-lineage ALL, was recognized as clonal in a T-lymphoblastic lymphoma (T-LL) case, and one TCRG clonal rearrangement was not previously identified because it was a very low represented clone (below the sensitivity of the homo/heteroduplex assay). Interestingly, the standard heteroduplex assessment failed to recognize two newly identified clonal rearrangements (*n* = 1 IGH and *n* = 1 IGK), despite their high clone abundance and the monoclonal feature. Additionally, two newly identified IGH bi-clonal rearrangements were not tested by the standard procedure because the paucity of the diagnostic bone marrow material did not allow for the performance of the complete conventional PCR panel. In the high-throughput approach, 600 ng of DNA was sufficient to perform the experiment and study the entire Ig/TCR region. In one of the two cases still lacking a clonal marker, the blasts percentage in the diagnostic bone marrow sample was around 5% (BG_39541).

On the basis of sequences provided by NGS, we were able to design allele-specific oligonucleotide (ASO)-qPCR assays for the MRD assessment. In cases for whom enough material was available (9 out of 10), we validated the assays on standard curve dilutions of diagnostic specimen in normal buffycoat. All the Ig/TCR rearrangements, including uncommon V-D-J combinations, proved useful as MRD markers. In detail, in five cases, we obtained at least one patient-specific assay with the required sensitivity of 10^−5^ and quantitative range of 10^−4^, confirming that the found rearrangements represented major leukemia clones missed by the conventional approach at diagnosis. For two patients, assays reached the sensitivity of 10^−4^ and the quantitative range of 10^−3^, while in the other two cases, the sensitivity was less than 10^−4^. Although low-sensitive assays are not useful to asses MRD negativity, they can identify MRD-positive patients, allowing appropriate medical intervention to avoid hematologic relapse. MRD assessment based on developed assays allows for the prompt allocation of one MRD-positive patient to stem cell transplant.

### 2.4. Gene Variant Analysis by NGS Approach

The single nucleotide variant (SNV) and insertion/deletion (indel) analysis of the genes of interest allowed for the identification of 51 gene variants in 18 out of 19 (94.7%) analyzed patients (5 B- and 14 T-lineage), involving the following genes: CREB Binding Protein (*CREBBP*), Enhancer Of Zeste 2 Polycomb Repressive Complex 2 Subunit (*EZH2*), F-Box And WD Repeat Domain Containing 7 (*FBXW7*), Fms Related Receptor Tyrosine Kinase 3 (*FLT3*), Isocitrate Dehydrogenase (NADP(+)) 2 (*IDH2*), IKAROS Family Zinc Finger 1 (*IKZF1*), Janus Kinase 1 (*JAK1*), Janus Kinase 2 (*JAK2*), Janus Kinase 3 (*JAK3*), KRAS Proto-Oncogene, GTPase (*KRAS*), Notch Receptor 1 (*NOTCH1*), NRAS Proto-Oncogene, GTPase (*NRAS*), Paired Box 5 (*PAX5*), Phosphatase And Tensin Homolog (*PTEN*), SH2B Adaptor Protein 3 (*SH2B3*), Tet Methylcytosine Dioxygenase 2 (*TET2*), and Tumor Protein P53 (*TP53*). The variant allele fraction (VAF) of the identified variants ranged from 5% to 79% (Table 4).

**Figure 1:**
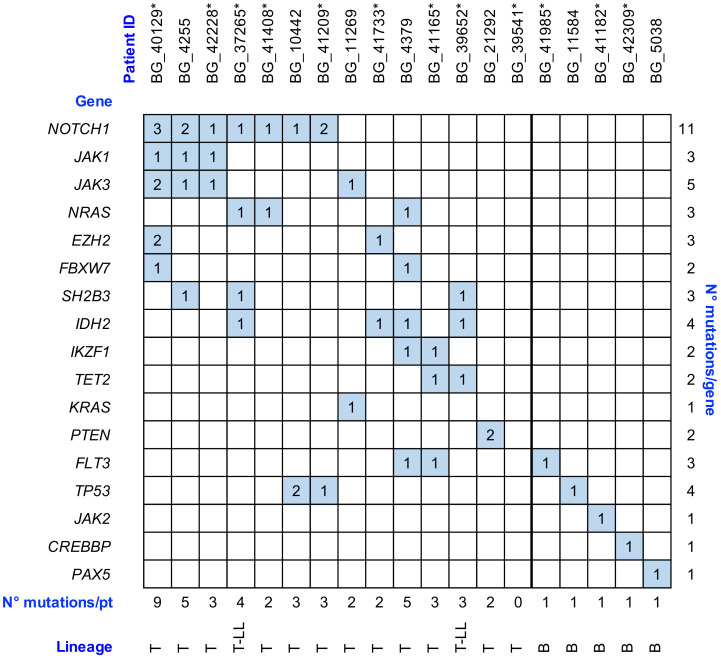
Summary of gene variants identified in 19 adult acute lymphoblastic leukemia (ALL)/lymphoblastic lymphoma (LL) patients. The filled boxes relate to the presence and the number of gene variants identified by capture-NGS. The 12 newly-diagnosed ALL/LL patients of the prospective cohort are indicated with “*”. For patient BG_39541, we detected no gene variants nor Ig/TCR rearrangements.

In all the B-ALL patients (BG_41985, BG_11584, BG_41182, BG_42309, and BG_5038), we identified a single variant involving one single gene (*FLT3*, *TP53, JAK2*, *CREBBP*, and *PAX5*, respectively), while only one T-ALL patient (BG_21292) presented a single gene involved. Interestingly, this T-ALL case harbored two mutations in the involved *PTEN* gene (Figure 1).

In the remaining 13 T-lineage-derived samples, we identified the presence of gene variants involving at least two genes and we observed a statistically significant correlation between T-lineage and the increase of mutations number per patient (*p* = 0.005). Concordantly with the literature, variants in *NOTCH1* were present in 7 out of 14 (50%) T-lineage patients [26,27] of the studied cohort and were associated with gene variants in *NRAS*, *TP53* or *JAK1*, and *JAK3*. No matter the co-occurrence with *NOTCH1* variants, *NRAS* mutations were located in the nucleotide-binding domain region of the protein. Furthermore, we observed a co-occurrence of variants in *JAK1*, *JAK3*, and *NOTCH1* genes in three different patients. In these cases, the *JAK1* gene harbored variants in the pseudokinase domain, while *JAK3* alterations, co-mutated with *JAK1* and *NOTCH1*, involved the JH1 kinase domain (p.L857P and p.D846N) or the linker region between the SH2 domain and the pseudokinase domain (p.M511I). This latter variant was previously described in T-cell malignancies as an activating amino acid change [28]. The *NOTCH1* variants associated with *JAK1* and *JAK3* mutations were located in the juxtamembrane heterodimerization (HD) N-terminal domain, but in one of these cases (BG_40129) we identified two additional variants of *NOTCH1* (p.P2514H and p.A2463PfsTer14) involving the domains PEST (a peptide sequence rich in proline (P), glutamic acid (E), serine (S), and threonine (T)) and TAD (transcriptional activation domain), respectively.

A statistical analysis performed on the co-occurrence of gene variants, although a limited number of patients were molecularly profiled, demonstrated a statistically significant association between *JAK1* and *JAK3* (*p* = 0.004) and *JAK1* and *NOTCH1* (*p* = 0.04). Moreover, in the T-LL studied samples, we identified the association of *IDH2* mutations and the presence of variants involving *SH2B3* (*p* = 0.09), while the co-occurrence of *FLT3* and *IKZF1* variants was observed in two out of three T-ALL cases. We did not observe any association between gene variants and specific Ig/TCR rearrangements. Of note, several variants (ranging from two to five) were identified in three patients in whom no Ig/TCR rearrangements were recognized.

## 3. Discussion

In this work, we developed and validated a novel capture-based NGS panel useful for Ig/TCR clonal marker screening and gene variant identification at diagnosis of ALL.

Although the identification process of leukemia-associated clonal Ig/TCR has been developed and ruled during the last 20 years within European collaborative networks (EuroClonality and EuroMRD, part of ESHLO foundation), this procedure remains laborious, time-consuming, and, in some cases, ineffective. Therefore, MRD stratification using the gold standard ASO-qPCR approach is nowadays feasible in about 95% of cases [12]. NGS technology has been demonstrated to be a powerful tool for the identification of clonal rearrangements at disease presentation, resulting in being less laborious and easier to perform than standard techniques [14,15,16]. Indeed, NGS proved faster compared to multistep standard workflow, allowing an efficient patient-specific probe design for timely MRD assessment, based on NGS-derived sequences.

Our capture-based NGS assay identified all clonal rearrangements previously found by standard PCR and allowed the additional identification of clonal markers that are difficult to detect by multiplex PCR [14]. It also provided Supplementary Information about bi-clonalities, oligoclonalities, and uncommon rearrangements. This latter aspect is particularly important in patients in whom no Ig/TCR clonalities can be identified by standard procedure. In our clinical study, which started in the year 2000, this MRD-unknown cohort represented 16% of the enrolled patients [5]. These patients could not have been followed during the treatment with an MRD patient-specific probe, allowing a treatment based only on clinical features that often proved not completely adequate to define the relapse risk of the patient. Therefore, we verified this new power of the assay on patients prospectively enrolled into the active trial in which the standard method failed. In this cohort for 9 out of 10 (90%) patients, a useful MRD assay has been designed on clonal sequences provided by capture-based NGS assay, thus allowing a prospective risk-class assignment (50%) or an MRD persistence identification. Moreover, NGS offers an important improvement in terms of increased sensitivity, resulting in the possibility to identify at diagnosis minor leukemia clones that could eventually be responsible for unexpected relapse. Indeed, the MRD monitoring by ASO-qPCR requires the choice of one or two clonal rearrangements for follow-up measurements [8,29], usually selected for better sensitivity and reflecting, in most cases, the abundance of the leukemic clone among the cell population. Therefore, the ASO-qPCR approach necessarily leads to losing track of minor subclones that might resist therapy and be responsible for disease resistance or reappearance [8]. Given this and considering our results, it could be desirable to explore all the possible rearrangements, including the uncommon ones not included into the standard PCR panel or amplicon-based NGS panel recently published [14]. Nowadays, some but not all lineage-specific rearrangements are investigated either in T- and B-lineage ALL with current methods. Indeed, it could be suitable to test all possible rearrangements in any ALL in order to avoid the missing of unexpected clonal markers.

A desirable approach to MRD assessment is also NGS-based. Some companies are offering a full MRD evaluation by receiving diagnostic and follow-up samples. The development of a standardized amplicon-based, open access method is still ongoing within collaborative groups [21] in which assay sensitivity and proper quantitation are the main issues. Upon resolving these issues, one point of weakness still should be faced, namely, the search of only a few, highly represented clones identified at diagnosis. This latter point would not be a problem for a capture-based method in which all the rearrangements are entirely studied in each experiment.

The capture-based NGS approach also proved useful for saving the diagnostic material collected at disease onset. Indeed, the amount of 1 μg of genomic DNA required for the complete NGS panel for clonal marker screening, including the study of rare rearrangements and gene profiling, is much lower than the amount of DNA needed for the amplicon-based techniques and the genetic profiling performed separately. This consideration is pivotal, especially from the perspective in which nowadays the MRD evaluation is still performed by ASO-qPCR, using serial dilutions of the diagnostic specimen, with MRD quantification being expressed as logarithmic reduction compared to the leukemia burden at disease onset. On the other hand, promising, newly described techniques such as amplicon NGS or droplet digital PCR (ddPCR), aiming to achieve absolute quantification of the residual leukemia level, are still under investigation and are not yet standardized [30]. Additionally, the saving of diagnostic material is essential in hypocellular specimens or when *punctio sicca* occurs and follow-up sample analysis is required for a long period of time.

In our work, all B-ALL MRD-unknown patients, tested retrospectively as well prospectively, showed at least one clonal rearrangement, while all patients still lacking a molecular marker in our cohort belonged to the T-lineage group. For two of these patients, we can speculate that the tested diagnostic sample was not adequate for the appropriate analysis. In fact, in one case (BG_4005), the blasts percent content in the diagnostic sample was not available and we cannot exclude that the specimen was not leukemia-representative. Likewise, the blasts percentage in the bone marrow diagnostic sample for patient BG_39541 was around 5%, not sufficient to the proper leukemia diagnosis according to the World Health Organization criteria. Even then, we can suppose that the available diagnostic specimen was not correctly evaluable. However, the results thus far obtained could suggest the definition of a small subgroup of very immature T-ALL in which Ig/TCR rearrangements had not yet occurred, although further analysis is required to speculate the maturation stage of leukemic lymphoblasts and to exclude technical limitations. Nevertheless, our NGS approach allowed us to identify very small leukemia clones, representing less than 1% of the global leukemia population; however, lacking a spike-in control to normalize clonal rearrangement size compared to physiological rearrangements in normal lymphocytes, we could not exclude a technical bias. Notably, the not-yet reported use as negative controls of HUVECs and mesenchymal cells revealed no generation of artificial rearrangements during library generation or bioinformatic data interpretation. In any case, it is important to note that the group of prospectively analyzed MRD-unknown patients is characterized by a strong prevalence of T-lineage cases, while globally the ratio between B- and T-ALL cases is unbalanced towards B-lineage occurrence.

The demanding process to obtain a mutational analysis of several genes that are prognostically significant [23] or defining leukemia cases with specific features (Philadelphia-like ALL) [25] prompted us to include a number of relevant genes into the capture design. This resulted in the identification of several mutations in the studied ALL cases. In particular, all the B-ALL cases harbored single gene variants involving *CREBBP*, *FLT3*, *JAK2*, *PAX5*, and *TP53.* On the contrary, in all the T-ALL-derived samples, we observed the presence of at least two gene variants, with a statistically significant increase of the mutations number in T-lineage patients compared to B-ALL cases. On the basis of the data thus far obtained, we can conclude that for T-ALL patients the inclusion of mutational status analysis of leukemia-associated genes could improve not only the general knowledge about leukemogenesis, but it could have important consequences in clinical practice. Indeed, we identified gene variants in three out of five T-ALL patients in which no informative Ig/TCR rearrangements were recognized. Two of these variants were druggable mutations (*IDH2* p.R140Q) that could be considered for therapeutic intervention.

Some limitations must be acknowledged. Our approach increases the complexity and the costs to identify clonal markers. Capture-based technology is not yet standardized and it should be externally validated before being routinely applied in clinical practice. Finally, this was a single center study in which only a limited number of patients were evaluated. A clinical multicenter validation was undertaken within the Euro-clonality NGS consortium [22].

## 4. Materials and Methods

### 4.1. Capture-Based NGS Panel Design

Two different capture-based NGS panels were designed using gene coordinates mapped on GRCh38/hg38 gene assembly, targeting coding V, D, and J genes in the Ig/TCR loci for the identification of D-J and V-D-J rearrangements (panel v1, 180 kb) and both Ig/TCR related regions, as well as a selected group of genes of interest listed in Table S1 (panel v2, 350 kb). No differences were detected between the two panels when applied to the same diagnostic sample (see Table S2, patient BG_5038) and no reduction in our ability to identify V-D-J rearrangements was observed. The mean of targeted aligned reads was 149,256 reads (min 228, max 276,500) for panel v1 and 2,391,017 reads (min 1893, max 4,392,496) for panel v2. The minimum analyzed (i.e., rearranged) sequences per sample should have been at least 1000.

### 4.2. Clinical Samples

Panel v1 was applied for the identification of clonal rearrangements on diagnostic bone marrow material of 10 adult ALL patients (6 B- and 4 T-lineage, median age 33 years), including 2 human umbilical vein endothelial cell (HUVEC) samples and 2 mesenchymal cord blood cell samples as negative controls for Ig/TCR rearrangements. These patients have been formerly studied for clonality assessment following the conventional EuroMRD guidelines [9,10], within the NILG-ALL 09/00 clinical trial (ClinicalTrials.gov identifier: NCT00358072) [5]. The same capture-based NGS approach for clonality assessment was applied on 23 adult ALL patients (13 B- and 10 T-lineage, median age 43 years) enrolled into the NILG-ALL 09/00 trial, in which the identification of a suitable MRD molecular marker with standard procedure failed (MRD-unknown). The NGS panel v2 was validated on diagnostic bone marrow samples of 7 adult ALL patients formerly evaluated for Ig/TCR rearrangements and analyzed for *TP53* mutational status by an independent NGS protocol (6 out of 7) [23]. The same panel was prospectively used on 12 newly diagnosed ALL patients (3 B-, 7 T-lineage, and 2 T-lymphoblastic lymphoma (T-LL); median age 40 years) missing suitable MRD assays by standard approach. Patients’ clinical and biological characteristics are reported in Tables S2 and S3. Since no oligoclonalities emerged from the analysis of capture-NGS performed on highly diluted diagnostic samples in normal polyclonal mononuclear cells (serial dilutions according to EuroMRD guidelines), we did not perform any specific tests to evaluate oligoclonalities in normal, polyclonal lymphocytes.

### 4.3. DNA Extraction, Library Preparation, and Sequencing

Genomic DNA was isolated by Gentra Puregene Cell Kit (Qiagen, Hilden, Germany) or Maxwell 16 Cell LEV DNA Purification Kit (Promega, Madison, WI, USA) from bone marrow mononuclear cells obtained by Ficoll gradient (Cedarlane Laboratories Ltd., Burlington, ON, Canada). For library preparation, we fragmented 600 ng–1 µg of genomic DNA to an average size of 600 bp by Covaris S220 Focused-Ultrasonicator (Covaris, Woburn, MA, USA) or Kapa enzymes (Kapa Biosystems, Wilmington, MA, USA). Libraries in the pre-hybridization steps were prepared with the KAPA HyperPlus Kit (Kapa Biosystems, Wilmington, MA, USA) and were then hybridized to our custom-designed EZ SeqCap gene panel (Roche NimbleGen, Madison, WI, USA). The pool of enriched libraries was sequenced on a MiSeq platform, using 300 bp paired-end reads (v3 chemistry, Illumina, San Diego, CA, USA).

### 4.4. NGS Data Analysis and Statistical Analysis

Bioinformatics data were processed separately. The identification of Ig/TCR rearrangements was performed through the open-source application Vidjil (www.vidjil.org) [31,32]. Uncommon V-D-J combinations were considered according to previously published literature [33,34,35,36]. Low clonal representation was stated when a V-(D)-J annotation with unique CDR3 region sequence was present with a clone size between 5% and 10% in the locus. Oligoclonality was defined when the same V-(D)-J annotation was present with 3 or more different CDR3 regions each having a clonal size ≥ 5%. The variant calling for gene alterations was performed by MiSeq Reporter Software, and visualization of results was achieved by Illumina VariantStudio Data Analysis Software (Illumina, San Diego, CA, USA) and the Integrative Genomics Viewer tool (http://software.broadinstitute.org/software/igv/) [37,38]. For gene variant analysis, we considered only variants with a read depth of at least 100 reads and VAF of 5%. Associations between different gene variants and between gene variants and other characteristics were assessed using the Fisher exact test for categorical variables and Wilcoxon rank-sum test for continuous variables. Two-sided *p*-values <0.1 were considered significant. Statistical analysis was performed with R software (version 3.6.2).

### 4.5. Clonality Assessment Validation

In all samples, we determined the Ig/TCR gene rearrangements at diagnosis by PCR, homo-heteroduplex clonality assay, and Sanger sequencing according to BIOMED protocols [9,10]. The finger-print CDR3 regions of V-D-J gene rearrangements were detailed through the IMGT V-Quest web application (www.IMGT.org). Additional rearrangements found by NGS were validated by BIOMED-1/2 primers, if available, or by custom-made PCR primers. The design of the latter was based on the Ig/TCR annotation derived from NGS sequences studied by IMGT; V-, D-, and J-specific primers were designed by Primer Express Software v2.0 (Applied Biosystems, Foster City, CA, USA) following BIOMED-1/2 recommendations. After PCR amplification, we performed homo-heteroduplex clonality assay and Sanger sequencing. In case of weak PCR product in heteroduplex analysis [10], we applied band elution and a second amplification before Sanger sequencing.

## 5. Conclusions

In conclusion, our results indicated that the prospective application of NGS remarkably simplified the assessment of clonality in adult ALL, providing additional information about the mutational status of some ALL-related genes, with it now being the new standard to identify the most informative Ig and TCR markers for the prospective evaluation of MRD.

Despite the long and extraordinarily successful history of Ig/TCR-based MRD evaluation for childhood [1,2,39,40] and adult ALL patients [3,4,5,41], we have to keep in mind that Ig/TCR are clonality markers and MRD evaluation reflects the leukemia chemo-sensitivity. No insight into leukemia development or targeted therapy can be drawn. This approach was developed in times in which the main information on ALL gene mutations was the broad gene involvement with low frequency and there was no technical support for the study of such a large number of genes. Nowadays, many new technologies have been developed that offer the possibility to analyze huge genome sections.

Our NGS approach including Ig/TCR rearrangements as well as mutations in an increasable number of genes could be a valid bridge toward a targeted MRD evaluation, allowing the accumulation of a solid, comparative, and prospective experience.

## Figures and Tables

**Table 1:** Immunoglobulin/T-cell receptor (Ig/TCR) rearrangements identified by next-generation sequencing (NGS) in the cohort of adult acute lymphoblastic leukemia (ALL) patients enrolled into the Northern Italy Leukemia Group (NILG)-ALL 09/00 clinical trial, formerly evaluated for clonality assessment. These rearrangements have been (Yes) or not (No) previously detailed by standard clonality assessment and Sanger sequencing following the conventional EuroMRD guidelines.

Patient ID	ALL Lineage	Locus	V Gene	del V	*n*	del D	D Gene	del D	*n*	del J	J Gene	Known	Rearrangement Feature
BG_371	B	IGH	IGHV3-9*01	−2	15	−2	IGHD6-19*01	−4	7	0	IGHJ3*02	Yes	/
		TRD+	TRDV2*02	−4	10	−28	TRDD3*01	NA	NA	NA	NA	Yes	/
		TRG	TRGV2*01	0	20	NA	NA	NA	NA	−3	TRGJ1*01	Yes	/
		TRG	TRGV3*02	−4	15	NA	NA	NA	NA	−6	TRGJ1*02	Yes	/
BG_4502	T	TRB	TRBV11-2*03	0	9	−3	TRBD2*01	−5	1	−4	TRBJ2-1*01	Yes	/
		TRB	TRBV14*02	−3	4	−1	TRBD2*01	NA	NA	NA	TRBJ2-6*01	Yes	/
		TRD	TRDV1*01	0	15	0	TRDD3*01	−2	1	−3	TRDJ1*01	Yes	/
		TRG	TRGV3*02	−1	10	NA	NA	NA	NA	−4	TRGJ1*02	Yes	/
		TRG	TRGV9*01	−1	13	NA	NA	NA	NA	−6	TRGJ1*02	Yes	/
BG_5038	B	IGH	IGHV3-49*02	−15	8	−2	IGHD2-8*01	0	2	−2	IGHJ6*02	Yes	/
		IGK	IGKV1-16*02	−17	9	−6	KDE	NA	NA	NA	NA	Yes	/
		TRB	TRBV20-1*05	0	7	−3	TRBD2*01	−5	3	−2	TRBJ2-3*01	Yes	/
		TRD+	TRDD2*01	−6	5	NA	NA	NA	NA	0	TRDD3*01	Yes	/
		TRG	TRGV9*01	0	5	NA	NA	NA	NA	−1	TRGJP1*01	Yes	/
		TRG	TRGV3*02	0	4	NA	NA	NA	NA	0	TRGJ1*02	Yes	/
BG_5418	B	IGH	IGHV4-30-2*01	−14	34	0	IGHD3-3*01	−7	8	−11	IGHJ6*02	Yes	/
		IGH	IGHV3-7*02	−1	2	−10	IGHD2-2*01	−5	6	−4	IGHJ6*02	Yes	/
		IGK	IGKV3-20*01	−3	3	NA	NA	NA	NA	0	KDE	Yes	/
		IGL	IGLV2-8*01	−9	8	NA	NA	NA	NA	0	IGLJ2*01	Yes	/
		TRG	TRGV9*01	−10	10	NA	NA	NA	NA	−5	TRGJ1*02	Yes	/
		TRG	TRGV11*01	−7	8	NA	NA	NA	NA	−3	TRGJ1*02	Yes	/
BG_5452	T	IGH	IGHV3-11*01	0	19	−2	IGHD6-19*01	−4	4	−9	IGHJ4*02	Yes	/
		IGH	IGHV3-64D*06	−2	33	2	IGHD6-19*01	−4	4	9	IGHJ4*02	Yes	/
		IGH	IGHV4-34*01	0	6	1	IGHD6-6*01	−7	1	0	IGHJ6*02	Yes	/
		IGH+	NA	NA	NA	NA	IGHD7-27*01	−2	3	−3	IGHJ2*01	Yes	/
		TRB	NA	NA	NA	NA	TRBD1	33	0	0	TRBJ2-3	Yes	/
		TRB	TRBV9*01	−3	10	NA	TRBD2*01	NA	NA	0	TRBJ2-1*01	Yes	/
		TRG	TRGV10*02	−2	11	NA	NA	NA	NA	−6	TRGJ1*02	Yes	/
		TRG	TRGV2*01	0	3	NA	NA	NA	NA	−1	TRGJ1*02	Yes	/
BG_9574	T	TRB	TRBV14*01	−1	11	−4	NA	NA	NA	NA	TRBJ2-7*01	Yes	/
		TRB	TRBV5-3*01	−2	3	0	TRBD1*01	−3	27	−4	TRBJ2-7*01	Yes	/
		TRD	TRDV1*01	−5	5	0	TRDD2*01	0	11	0	TRDJ1*01	Yes	/
		TRG	TRGV11*01	−11	3	−2	NA	NA	NA	NA	TRGJ2*01	Yes	/
		TRG	TRGV4*02	−6	2	−1	NA	NA	NA	NA	TRGJ1*02	Yes	/
		TRG	TRGV8*01	−1	3	−3	NA	NA	NA	NA	TRGJP2*01	Yes	/
BG_9813	T	TRB+	NA	NA	NA	NA	TRBD2*01	−3	0	−5	TRBJ2-5*01	Yes	/
BG_9813	T	TRD+	TRDD2*01	−10	22	NA	NA	NA	NA	0	TRDJ1*01	Yes	/
		TRG	TRGV10*02	−1	3	NA	NA	NA	NA	−6	TRGJ1*02	Yes	/
		TRG	TRGV9*01	−3	3	NA	NA	NA	NA	0	TRGJ2*01	Yes	/
BG_11360	B	IGH	IGHV4-4*07	−2	9	−1	IGHD2-15*01	−6	12	−30	IGHJ6*04	Yes	/
		IGH+	NA	NA	NA	NA	IGHD7-27*01	0	12	−7	IGHJ6*02	Yes	/
		TRD+	TRDD2*01	0	5	NA	NA	NA	NA	−1	TRDD3*01	Yes	/
		TRD+	TRDV2*02	0	13	NA	NA	NA	NA	−3	TRDD3*01	Yes	/
BG_11720	B	IGH	IGHV6-1*01	0	5	−3	IGHD3-3*01	−2	2	−6	IGHJ6*02	Yes	/
		TRA+D	TRDV2*03	0	5	−2	TRDD3*01	0	3	−4	TRAJ9*01	Yes	/
		TRG	TRGV9*01	−6	13	NA	NA	NA	NA	−1	TRGJ1*02	Yes	/
BG_11806	B	IGH	IGHV1-2*02	−1	3	−3	IGHD2-21*02	−3	24	−1	IGHJ5*02	Yes	/
		IGK	IGKV2-28*01	0	6	NA	NA	NA	NA	−1	IGKJ4*02	Yes	/
		IGL	IGLV3-19*01	−5	14	NA	NA	NA	NA	−4	IGLJ3*02	Yes	/
		TRD+	TRDD2*01	0	2	NA	NA	NA	NA	0	TRDD3*01	Yes	/
		TRG	TRGV5*01	0	3	NA	NA	NA	NA	−1	TRGJ1*02	Yes	/
BG_11806	B	TRD+	TRDD2*01	0	13	NA	NA	NA	NA	0	TRDJ3*01	No	Uncommon V/DJ combinations
		TRA+D	TRDD2*01	−2	3	NA	NA	NA	NA	−7	TRAJ30*01	No	Uncommon V/DJ combinations
BG_5038	B	TRA+D	TRDD2*01	−3	53	NA	NA	NA	NA	−15	TRAJ48*01	No	Uncommon V/DJ combinations
BG_5418	B	TRA+D	TRDD2*01	−1	14	NA	NA	NA	NA	−5	TRAJ29*01	No	Uncommon V/DJ combinations
		TRA+D	TRDD2*01	−11	4	NA	NA	NA	NA	−1	TRAJ23*01	No	Uncommon V/DJ combinations
		TRA	TRAV26-1*01	0	4	NA	NA	NA	NA	−2	TRAJ33*01	No	Uncommon V/DJ combinations
		TRA	TRAV8-3*01	0	8	NA	NA	NA	NA	−7	TRAJ34*01	No	Uncommon V/DJ combinations
BG_5452	T	TRA	TRAV21*01	0	1	NA	NA	NA	NA	0	TRAJ48*01	No	Uncommon V/DJ combinations
BG_9574	T	TRA+D	TRAV29/DV5*01	0	38	0	TRDD3*01	−4	3	0	TRDJ1*01	No	Uncommon V/DJ combinations
		TRA	TRAV21*02	−7	0	−5	NA	NA	NA	NA	TRAJ24*02	No	Uncommon V/DJ combinations
BG_9813	T	TRA	TRAV19*01	−2	10	NA	NA	NA	NA	−4	TRAJ36*01	No	Uncommon V/DJ combinations
BG_11360	B	IGH	IGHV4-34*01	0	9	−18	IGHD2-2*01	0	2	−4	IGHJ6*03	No	Oligoclonality
BG_11720	B	IGH	IGHV4-34*01	0	6	−1	IGHD6-6*01	−7	1	0	IGHJ6*02	No	Oligoclonality
BG_371	B	IGK	IGKV1-33*01	−1	2	NA	NA	NA	NA	−2	IGKJ4*01	No	Oligoclonality
		IGK	IGKV1-39*01	−4	8	NA	NA	NA	NA	−9	IGKJ2*02	No	Oligoclonality
		TRG	TRGV4*02	−4	5	NA	NA	NA	NA	−3	TRGJ1*01	No	Oligoclonality
BG_5418	B	TRB	TRBV23-1*01	0	1	0	TRBD2*01	−7	14	−6	TRBJ2-7*01	No	Oligoclonality
		TRB	TRBV10-3*01	−8	18	−7	TRBD2*01	NA	NA	NA	TRBJ2-3*01	No	Oligoclonality
		TRB	TRBV24-1*01	−13	5	−2	TRBD1*01	−3	5	2	TRBJ2-7*01	No	Oligoclonality
BG_5452	T	IGH	IGHV6-1*01	0	5	−3	IGHD3-3*01	−2	2	−6	IGHJ6*02	No	Oligoclonality
BG_9574	T	TRG	TRGV11*01	0	1	−25	NA	NA	NA	NA	TRGJ1*02	No	Oligoclonality
BG_11360	B	IGL	IGLV3-10*01	−2	7	NA	NA	NA	NA	0	IGLJ3*02	No	Low represented clone
BG_371	B	IGH	IGHV1-3*02	−2	4	−17	IGHD3-16*01	−15	0	−18	IGHJ4*02	No	Low represented clone
BG_9813	T	TRB	TRBV4-3*01	−1	21	−6	TRBD2*02	NA	NA	NA	TRBJ1-1*01	No	Low represented clone

IGK: IGκ rearrangements, IGL: IGλ rearrangements, TRB: TCRβ rearrangements, TRG: TCRγ rearrangements, TRD: TCRδ rearrangements, and TRA: TCRα rearrangements. Symbol “+” refers to incomplete rearrangements. del V: nucleotides deletion in V gene segment, *n*: number of inserted nucleotides, del D: nucleotides deletion in D gene segment, del J: nucleotides deletion in J gene segment, NA: not applicable, Yes: rearrangement already identified by standard procedure, No: rearrangement identified by NGS and not previously identified by standard procedure.

**Table 2:** Ig/TCR rearrangements identified by NGS in the minimal residual disease (MRD)-unknown cohort of adult ALL patients enrolled into the NILG-ALL 09/00 clinical trial. These rearrangements have not been previously identified by standard clonality assessment and Sanger sequencing following the conventional EuroMRD guidelines, or they were not adequate to generate a suitable patient-specific molecular probe for the MRD evaluation.

Patient ID	ALL Lineage	Locus	V Gene	del V	*n*	del D	D Gene	del D	*n*	del J	J Gene	Validation	Rearrangement Feature
BG_11584	B	IGH	IGHV6-1*01	−2	8	−6	IGHD6-6*01	0	3	−10	IGHJ4*02	Yes	/
BG_2097	B	IGH	NA	NA	NA	NA	IGHD4-23*01	−2	1	−4	IGHJ2*01	Yes	/
BG_855	B	IGH	IGHV6-1*01	−3	2	−4	IGHD2-2*01	−1	5	0	IGHJ6*03	Yes	/
		IGH	IGHV3-23*01	0	19	−2	IGHD3-9*01	−8	13	−7	IGHJ4*02	Yes	/
BG_10112	B	IGH+	NA	NA	NA	NA	IGHD6-6*01	−2	0	−7	IGHJ4*02	Yes	/
		IGH+	NA	NA	NA	NA	IGHD1-7*01	−6	2	−4	IGHJ4*02	Yes	/
BG_11053	B	IGH+	NA	NA	NA	NA	IGHD2-2*01	−6	3	−4	IGHJ4*02	Yes	/
BG_1125	B	IGH+	NA	NA	NA	NA	IGHD6-25*01	0	−5	11	IGHJ4*02	Yes	/
BG_11584	B	IGH+	NA	NA	NA	NA	IGHD1-26*01	0	3	−3	IGHJ3*02	Yes	/
BG_4254	B	IGH+	NA	NA	NA	NA	IGHD3-9*01	−5	4	−11	IGHJ6*03	Yes	/
		IGH+	NA	NA	NA	NA	IGHD6-6*01	−4	3	−15	IGHJ5*02	Yes	/
BG_5702	B	IGH+	NA	NA	NA	NA	IGHD2-2*02	−6	7	−5	IGHJ5*02	Yes	/
BG_8345	B	IGH+	NA	NA	NA	NA	IGHD3-22*01	−2	7	1	IGHJ6*02	Yes	/
		IGH+	NA	NA	NA	NA	IGHD1-26*01	−2	0	7	IGHJ4*02	Yes	/
BG_9445	B	IGH+	NA	NA	NA	NA	IGHD2-2*02	−3	19	5	IGHJ6*03	Yes	/
BG_11269	T	TRB	TRBV7-8*01	−3	14	NA	NA	NA	NA	−7	TRBJ1-4*01	Yes	/
BG_6037	T	TRB	TRBV4-2*01	0	25	NA	NA	NA	NA	−3	TRBJ2-3*01	Yes	/
		TRB	TRBV20-1*02	−3	15	−2	TRBD2*02	−2	19	−6	TRBJ2-1*01	Yes	/
BG_10112	B	TRG	TRGV11*01	−4	7	NA	NA	NA	NA	−8	TRGJP1*01	Yes	/
		TRG	TRGV11*01	−3	10	NA	NA	NA	NA	−1	TRGJ2*01	Yes	/
BG_11269	T	TRG	TRGV4*02	−4	5	NA	NA	NA	NA	0	TRGJ2*01	Yes	/
		TRG	TRGV10*02	−6	0	NA	NA	NA	NA	−8	TRGJ1*02	Yes	/
BG_11584	B	TRG	TRGV4*02	−6	2	NA	NA	NA	NA	0	TRGJ2*01	Yes	/
		TRG	TRGV10*02	0	NA	NA	NA	NA	4	−9	TRGJP1*01	Yes	/
		TRG	TRGV11*01	0	0	NA	NA	NA	NA	−7	TRGJP1*01	Yes	/
BG_855	B	TRG	TRGV9*01	−10	3	NA	NA	NA	NA	−2	TRGJ1*02	Yes	/
BG_11053	B	TRD+	NA	NA	NA	NA	TRDD2*01	−1	22	−1	TRDJ1*01	Yes	/
		TRD+	NA	NA	NA	NA	TRDD2*01	0	19	0	TRDJ4*01	Yes	/
BG_4254	B	TRD+	NA	NA	NA	NA	TRDD2*01	0	5	−2	TRDD3*01	Yes	/
BG_8345	B	TRD+	NA	NA	NA	NA	TRDD2*01	−3	15	0	TRDD3*01	Yes	/
BG_11053	B	IGH+	NA	NA	NA	NA	IGHD3-9*01	−5	3	−4	IGHJ4*02	Yes	Oligoclonality
		IGH+	NA	NA	NA	NA	IGHD3-9*01	−5	0	−4	IGHJ6*02	Yes	Oligoclonality
		IGH+	NA	NA	NA	NA	IGHD3-9*01	−3	13	−6	IGHJ6*02	Yes	Oligoclonality
BG_11345	T	TRD+	NA	NA	NA	NA	TRDD2*01	−8	0	−14	TRDD3*01	Yes	Oligoclonality
BG_1125	B	IGK	IGKV1-27*01	−1	0	NA	NA	NA	NA	2	IGKJ1*01	YesŦ	/
BG_11269	T	IGK	IGKV4-1*01	−1	0	NA	NA	NA	NA	−4	IGKJ4*01	YesŦ	/
		IGK	IGKV2D-29*01	−2	6	NA	NA	NA	NA	0	IGKJ2*01	YesŦ	/
		IGK	IGKV1-6*01	−3	0	NA	NA	NA	NA	0	IGKJ1*01	YesŦ	/
BG_6037	T	TRG	TRGV3*01	0	0	NA	NA	NA	NA	0	TRGJ1*02	YesŦ	/
		TRG	TRGV2*01	0	4	NA	NA	NA	NA	−2	TRGJ1*02	YesŦ	/
BG_2097	B	TRA+D	TRDV1*01	−2	8	NA	NA	NA	NA	−8	TRAJ29*01	YesŦ	/
BG_855	B	TRA+D	NA	NA	NA	NA	TRDD2*01	−4	24	−5	TRAJ29*01	YesŦ	/
		TRA+D	TRDV2*01	0	6	0	TRDD3*01	0	5	−4	TRAJ58*01	YesŦ	/
BG_11269	T	TRA	TRAV26-1*01	−3	3	NA	NA	NA	NA	−4	TRAJ4*01	YesŦ	/
BG_12438	T	TRA	TRAV19*01	−8	4	NA	NA	NA	NA	0	TRAJ47*02	YesŦ	/
BG_2097	B	TRA	TRAV13-1*01	−3	0	NA	NA	NA	NA	−3	TRAJ35*01	YesŦ	/
BG_5702	B	TRA	TRAV16*01	−6	2	NA	NA	NA	NA	−4	TRAJ9*01	YesŦ	/
BG_10487	B	IGH+	NA	NA	NA	NA	IGHD3-16*02	−5	8	0	IGHJ4*02	YesŦ	Biclonal sequence
		IGH+	NA	NA	NA	NA	IGHD3-3*01	0	11	−3	IGHJ5*02	YesŦ	Biclonal sequence
BG_6490	B	IGH+	NA	NA	NA	NA	IGHD2-21*02	−1	2	0	IGHJ6*03	YesŦ	Biclonal sequence
		IGH+	NA	NA	NA	NA	IGHD2-8*01	−7	8	−2	IGHJ3*02	YesŦ	Biclonal sequence
BG_8646	T	TRD+	NA	NA	NA	NA	TRDD2*01	−8	0	−14	TRDD3*01	YesŦ	Biclonal sequence
		TRD+	NA	NA	NA	NA	TRDD2*01	−8	0	−16	TRDD3*01	YesŦ	Biclonal sequence
BG_1125	B	IGH	IGHV4-34*02	0	10	NA	NA	NA	NA	−4	IGHJ5*01	No	Low represented clone
BG_5702	B	IGK	IGKV2-28*01	−3	2	−7	KDE	NA	NA	NA	NA	No	Low represented clone
BG_12438	T	TRB	TRBV4-1*02	−3	2	−1	TRBD1*01	−3	8	−2	TRBJ2-7*01	No	Low represented clone
BG_5702	B	TRB	TRBV4-1*02	0	2	0	TRBD1*01	−4	10	−8	TRBJ1-1*01	No	Low represented clone
BG_11053	B	TRG	TRGV9*01	−1	6	NA	NA	NA	NA	0	TRGJ1*02	No	Low represented clone
BG_3895	T	TRG	TRGV2*01	0	4	NA	NA	NA	NA	−10	TRGJP2*01	No	Low represented clone
BG_11053	B	TRD+	NA	NA	NA	NA	TRDD2*01	0	14	−10	TRDJ1*01	No	Low represented clone
BG_12438	T	TRA	TRAV21*01	−4	7	NA	NA	NA	NA	0	TRAJ27*01	No	Low represented clone
BG_2097	B	TRA	TRAV19*01	−10	5	NA	NA	NA	NA	0	TRAJ47*01	No	Low represented clone
BG_6490	B	IGH	IGHV4-34*12	0	9	1	IGHD2-8*02	−7	8	−2	IGHJ3*02	No	Oligoclonality
BG_5702	B	IGK	IGKV3-20*01	−4	6	NA	NA	NA	NA	−2	IGKJ2*01	No	Oligoclonality
BG_2097	B	TRB	TRBV6-5*01	0	2	−4	TRBD2*01	−7	0	−4	TRBJ1-5*01	No	Oligoclonality
BG_11053	B	TRG	TRGV4*02	−3	0	NA	NA	NA	NA	−4	TRGJ2*01	No	Oligoclonality
		TRG	TRGV11*01	−7	7	NA	NA	NA	NA	−1	TRGJ2*01	No	Oligoclonality
BG_2097	B	TRG	TRGV3*01	0	15	NA	NA	NA	NA	−1	TRGJ2*01	No	Oligoclonality
BG_9445	B	TRG	TRGV10*02	0	3	NA	NA	NA	NA	4	TRGJ1*01	No	Oligoclonality
BG_11584	B	IGH	IGHV4-31*02	−1	1	−3	IGHD3-10*01	−6	0	0	IGHJ4*02	ND	<5%
BG_2481	T	IGH	NA	NA	NA	NA	IGHD7-27*01	−11	0	0	IGHJ1*01	ND	<5%
BG_11054	B	IGH+	NA	NA	NA	NA	IGHD6-13*01	3	0	21	IGHJ1*01	ND	<5%
		IGH+	NA	NA	NA	NA	IGHD7-27*01	0	2	6	IGHJ4*02	ND	<5%
BG_10112	B	TRG	TRGV5*01	0	6	NA	NA	NA	NA	−1	TRGJ1*02	ND	<5%
		TRG	TRGV9*01	0	9	NA	NA	NA	NA	0	TRGJ1*02	ND	<5%
		TRG	TRGV11*01	−9	12	NA	NA	NA	NA	−5	TRGJ1*02	ND	<5%
		TRG	TRGV9*01	−2	4	NA	NA	NA	NA	0	TRGJ2*01	ND	<5%
		TRG	TRGV9*01	0	4	NA	NA	NA	NA	−2	TRGJ2*01	ND	<5%
BG_11053	B	TRG	TRGV10*02	−2	0	NA	NA	NA	NA	−1	TRGJ2*01	ND	<5%
		TRG	TRGV11*01	−2	15	NA	NA	NA	NA	0	TRGJ1*02	ND	<5%
		TRG	TRGV11*01	0	10	NA	NA	NA	NA	−8	TRGJ1*01	ND	<5%
		TRG	TRGV5*01	0	3	NA	NA	NA	NA	−5	TRGJ1*02	ND	<5%
BG_11584	B	TRG	TRGV11*01	0	NA	NA	NA	NA	5	−2	TRGJ1*02	ND	<5%
		TRG	TRGV11*01	−7	NA	NA	NA	NA	3	−7	TRGJP1*01	ND	<5%
		TRG	TRGV9*01	−2	NA	NA	NA	NA	11	−8	TRGJ1*01	ND	<5%
		TRG	TRGV11*01	−3	NA	NA	NA	NA	0	−16	TRGJ1*02	ND	<5%
BG_2097	B	TRG	TRGV4*02	0	6	NA	NA	NA	NA	−5	TRGJP1*01	ND	<5%
		TRG	TRGV4*01	−5	14	NA	NA	NA	NA	0	TRGJ1*02	ND	<5%
BG_11053	B	TRD+	NA	NA	NA	NA	TRDD2*01	0	16	0	TRDJ1*01	ND	<5%
BG_12438	T	TRG	TRGV8*01	1	0	NA	NA	NA	NA	−7	TRGJP2*01	ND	ND
BG_10640	B	IGH+	NA	NA	NA	NA	IGHD3-3*01	−4	6	−5	IGHJ6*02	ND•	ND
		IGH+	NA	NA	NA	NA	IGHD2-2*02	−3	4	−10	IGHJ6*02	ND•	ND
BG_4005	T	Na	Na	Na	Na	Na	Na	Na	Na	Na	Na	ND	/
BG_4255	T	Na	Na	Na	Na	Na	Na	Na	Na	Na	Na	ND	/
BG_4379	T	Na	Na	Na	Na	Na	Na	Na	Na	Na	Na	ND	/

For patients BG_4005, BG_4255, and BG_4379, we detected no Ig/TCR rearrangements. IGK: IGκ rearrangements, IGL: IGλ rearrangements, TRB: TCRβ rearrangements, TRG: TCRγ rearrangements, TRD: TCRδ rearrangements, and TRA: TCRα rearrangements. Symbol “+” refers to incomplete rearrangements. del V: nucleotides deletion in V gene segment, *n*: number of inserted nucleotides, del D: nucleotides deletion in D gene segment, del J: nucleotides deletion in J gene segment, NA: not applicable, Na: not available, Yes: rearrangement confirmed by standard procedure, Ŧ: use of specific primers for conventional PCR set-up required, No: rearrangement identified by NGS not validated by standard procedure, ND: not done, ND•: not done/exhausted diagnostic material.

**Table 3:** Ig/TCR rearrangements identified by NGS in the MRD-unknown cohort of newly diagnosed adult ALL patients. These rearrangements have not been previously identified by standard clonality assessment and Sanger sequencing following the conventional EuroMRD guidelines, or they were not adequate to generate a suitable patient-specific molecular probe for the MRD evaluation.

Patient ID	ALL lineage	Locus	V Gene	del V	*n*	del D	D Gene	del D	*n*	del J	J Gene	Known	Rearrangement feature
BG_41182	B	TRG	TRGV9*01	−1	0	NA	NA	NA	NA	−8	TRGJ1*02	Yes	/
		TRG	TRGV9*01	−2	3	NA	NA	NA	NA	0	TRGJ2*01	Yes	/
BG_41408	T	TRB	TRBV6-1*01	−10	26	NA	NA	NA	NA	0	TRBJ2-1*01	Yes	/
		TRG	TRGV11*01	−2	8	NA	NA	NA	NA	0	TRGJP2*01	Yes	/
		TRG	TRGV2*01	−25	0	NA	NA	NA	NA	0	TRGJP2*01	Yes	/
		TRD	TRDV2*02	−4	31	NA	NA	NA	NA	0	TRDJ1*01	Yes	/
BG_41985	B	TRG	TRGV2*02	−4	5	NA	NA	NA	NA	−7	TRGJ1*02	Yes	/
		TRG	TRGV3*01	−1	5	NA	NA	NA	NA	0	TRGJP2*01	Yes	/
		TRG	TRGV10*02	−1	6	NA	NA	NA	NA	−8	TRGJ1*02	Yes	/
BG_42228	T	TRB+	NA	NA	NA	NA	TRBD2*01	−2	1	−5	TRBJ2-7*01	Yes	/
		TRG	TRGV2*02	0	4	NA	NA	NA	NA	−2	TRGJ1*02	Yes	/
		TRG	TRGV2*01	0	6	NA	NA	NA	NA	−9	TRGJ1*02	Yes	/
BG_42309	B	IGH	IGHV3-23*01	−4	19	−9	IGHD3/OR15-3a*01	−9	8	−11	IGHJ6*02	Yes	/
		IGK+	Intron	0	2	NA	NA	NA	NA	−5	KDE	Yes	/
BG_41985	B	IGH+	NA	NA	NA	NA	IGHD1-7*01	0	1	−2	IGHJ6*02	ND•	/
		IGH+	NA	NA	NA	NA	IGHD1-7*01	−3	11	−17	IGHJ6*02	ND•	/
BG_41165	T	TRB	TRBV30*02	−2	10	NA	NA	NA	NA	0	TRBJ1-4*01	Na	Uncommon V/DJ combinations and low represented clone
		TRA	TRAV19*01	−2	4	NA	NA	NA	NA	−3	TRAJ50*01	Na	Uncommon V/DJ combinations and low represented clone
BG_41408	T	TRD	TRDV1*01	−8	20	0	TRDD3*01	−3	5	−3	TRDJ4*01	Na	Uncommon V/DJ combinations
BG_42228	T	TRD	TRDV3*01	−1	9	0	TRDD3*01	−1	0	−3	TRDJ1*01	Na	Uncommon V/DJ combinations
		TRA	TRAV21*01	−4	1	NA	NA	NA	NA	−1	TRAJ29*01	Na	Uncommon V/DJ combinations
BG_42309	B	IGK	GKV3-15*01	0	3	NA	NA	NA	NA	−1	IGKJ2*03	Na	Uncommon V/DJ combinations
		IGL	IGLV2-23*01	0	1	NA	NA	NA	NA	0	IGLJ3*02	Na	Uncommon V/DJ combinations
BG_37265	T-LL	IGK+	IGKV3-15*01	−2	5	NA	NA	NA	NA	6	KDE	Na	Uncommon and NT in T-lineage
		IGK+	IGKV2-28*01	−3	0	NA	NA	NA	NA	0	IGKJ4*01	Na	Uncommon and NT in T-lineage
		IGK+	IGKV1-33*01	−3	0	NA	NA	NA	NA	0	IGKJ4*01	Na	Uncommon and NT in T-lineage
BG_41182	B	TRA+D	NA	NA	NA	NA	TRDD2*01	−3	7	−3	TRAJ48*01	No-Na	Uncommon V/DJ combinations
		IGL	IGLV4-3*01	0	2	NA	NA	NA	NA	−2	IGLJ3*02	No	Uncommon V/DJ combinations
		IGL	IGLV3-1*01	−4	3	NA	NA	NA	NA	−5	IGLJ2*01	No	Uncommon V/DJ combinations
		TRG	TRGV10*02	−19	8	NA	NA	NA	NA	−17	TRGJP1*01	No	Uncommon V/DJ combinations
BG_41408	T	TRB	TRBV9*01	−4	15	NA	NA	NA	NA	0	TRBJ2-1*01	No	Oligoclonality
BG_40129	T	TRD+	TRDV2*01	0	2	NA	NA	NA	NA	0	TRDD3*01	No	Missed by heteroduplex
BG_41182	B	IGH	IGHV3-38-3*01	11	9	NA	NA	NA	NA	2	IGHJ6*03	No	Missed by heteroduplex
BG_41209	T	TRD+	NA	NA	NA	NA	TRDD2*01	−7	4	−13	TRDD3*01	No	Missed by heteroduplex
BG_42228	T	TRB	TRBV4-1*02	0	4	0	TRBD2*01	−2	0	−5	TRBJ2-1*01	No	Missed by heteroduplex
BG_42309	B	IGK+	IGKV1-17*01	−1	0	NA	NA	NA	NA	8	KDE	No	Missed by heteroduplex
BG_39652	T-LL	TRG	TRGV4*01	−2	0	NA	NA	NA	NA	−5	TRGJ1*01	No	Low represented clone
BG_41165	T	TRG	TRGV4*01	0	4	NA	NA	NA	NA	−11	TRGJ1*02	No	Low represented clone
		TRG	TRGV11*01	−11	17	NA	NA	NA	NA	6	TRGJP*01	No	Low represented clone
BG_41182	B	TRG	TRGV2*01	−3	2	NA	NA	NA	NA	−8	TRGJ1*02	No	Low represented clone
BG_41733	T	Na	Na	Na	Na	Na	Na	Na	Na	Na	Na	ND	/
BG_9541	T	Na	Na	Na	Na	Na	Na	Na	Na	Na	Na	ND	/

For patients BG_41733 and BG_9541, no Ig/TCR rearrangements were detected. T-LL: T-lymphoblastic lymphoma, IGK: IGκ rearrangements, IGL: IGλ rearrangements, TRB: TCRβ rearrangements, TRG: TCRγ rearrangements, TRD: TCRδ rearrangements, and TRA: TCRα rearrangements. Symbol “+” refers to incomplete rearrangements. del V: nucleotides deletion in V gene segment, *n*: number of inserted nucleotides, del D: nucleotides deletion in D gene segment, del J: nucleotides deletion in J gene segment, NA: not applicable, Na: not available, Yes: rearrangement already identified by standard procedure, No: rearrangement identified by NGS and not previously identified by standard procedure, ND: not done, ND•: not done/exhausted diagnostic material.

**Table 4:** List of gene variants identified in 18 out of 19 adult ALL patients of which 7 were formerly evaluated for Ig/TCR rearrangements and 12 were newly-diagnosed ALL patients.

Patient ID	Gene	HGVSc	HGVSp	VAF	RD	ARD	Consequence
BG_42309	*CREBBP*	NM_004380.2:c.4427C>T	NP_004371.2:p.Pro1476Leu	78.6	641	504	missense_variant
BG_40129	*EZH2*	NM_004456.4:c.1613C>T	NP_004447.2:p.Ser538Leu	51.7	410	212	missense_variant
BG_40129	*EZH2*	NM_004456.4:c.347T>C	NP_004447.2:p.Leu116Pro	43.3	282	122	missense_variant
BG_41733	*EZH2*	NM_004456.4:c.1987T>A	NP_004447.2:p.Tyr663Asn	18.2	292	53	missense_variant
BG_40129	*FBXW7*	NM_033632.3:c.1513C>T	NP_361014.1:p.Arg505Cys	7.4	542	40	missense_variant
BG_4379	*FBXW7*	NM_033632.3:c.62G>A	NP_361014.1:p.Gly21Asp	47.7	2514	1200	missense_variant
BG_41165	*FLT3*	NM_004119.2:c.1779_1793dupTTTCAGAGAATATGA	NP_004110.2:p.Asp593_Tyr597dup	11.8	330	39	inframe_insertion
BG_41985	*FLT3*	NM_004119.2:c.2503G>A	NP_004110.2:p.Asp835Asn	27.6	181	50	missense_variant
BG_4379	*FLT3*	NM_004119.2:c.2864A>G	NP_004110.2:p.Tyr955Cys	51.7	1412	730	missense_variant
BG_37265	*IDH2*	NM_002168.2:c.419G>A	NP_002159.2:p.Arg140Gln	22.7	1122	255	missense_variant
BG_39652	*IDH2*	NM_002168.2:c.547delGACinsAAG	NP_002159.2:p.Asp183Lys	5.6	144	8	missense_variant
BG_41733	*IDH2*	NM_002168.2:c.419G>A	NP_002159.2:p.Arg140Gln	45.8	690	316	missense_variant
BG_4379	*IDH2*	NM_002168.2:c.419G>A	NP_002159.2:p.Arg140Gln	42.9	1559	668	missense_variant
BG_41165	*IKZF1*	NM_006060.4_dupl12.1:c.849G>T	NP_006051.1_dupl12.1:p.Arg284Leu	43.6	165	72	missense_variant
BG_4379	*IKZF1*	NM_006060.4_dupl12.1:c.396G>T	NP_006051.1_dupl12.1:p.Gly133Val	49.0	514	252	missense_variant
BG_40129	*JAK1*	NM_002227.2:c.1954T>C	NP_002218.2:p.Tyr652His	52.5	179	94	missense_variant
BG_42228	*JAK1*	NM_002227.2:c.2107A>T	NP_002218.2:p.Ser703Cys	42.8	566	242	missense_variant
BG_4255	*JAK1*	NM_002227.2:c.2170C>T	NP_002218.2:p.Arg724Cys	12.2	1199	146	missense_variant
BG_41182	*JAK2*	NM_004972.3:c.2171T>C	NP_004963.1:p.Ile724Thr	12.0	465	56	missense_variant
BG_11269	*JAK3*	NM_000215.3:c.1370G>A	NP_000206.2:p.Cys457Tyr	49.2	177	87	missense_variant
BG_40129	*JAK3*	NM_000215.3:c.2570T>C	NP_000206.2:p.Leu857Pro	47.2	301	142	missense_variant
BG_40129	*JAK3*	NM_000215.3:c.2536G>A	NP_000206.2:p.Asp846Asn	13.4	307	41	missense_variant
BG_42228	*JAK3*	NM_000215.3:c.1533G>A	NP_000206.2:p.Met511Ile	44.0	650	286	missense_variant
BG_4255	*JAK3*	NM_000215.3:c.1533G>A	NP_000206.2:p.Met511Ile	29.1	955	278	missense_variant
BG_11269	*KRAS*	NM_033360.2:c.182A>T	NP_203524.1:p.Gln61Leu	6.6	802	53	missense_variant
BG_10442	*NOTCH1*	NM_017617.3:c.7324_7325insTC	NP_060087.3:p.Asp2442ValfsTer36	31.3	412	129	frameshift_variant
BG_37265	*NOTCH1*	NM_017617.3:c.5033T>C	NP_060087.3:p.Leu1678Pro	5.8	291	17	missense_variant
BG_40129	*NOTCH1*	NM_017617.3:c.4799T>A	NP_060087.3:p.Leu1600Gln	43.8	105	46	missense_variant
BG_40129	*NOTCH1*	NM_017617.3:c.7541C>A	NP_060087.3:p.Pro2514His	28.6	276	79	missense_variant
BG_40129	*NOTCH1*	NM_017617.3:c.7387delG	NP_060087.3:p.Ala2463ProfsTer14	5.8	223	13	frameshift_variant
BG_41209	*NOTCH1*	NM_017617.3:c.5165A>C	NP_060087.3:p.Gln1722Pro	24.7	174	43	missense_variant, splice_region_variant
BG_41209	*NOTCH1*	NM_017617.3:c.4787T>C	NP_060087.3:p.Leu1596Pro	6.0	117	7	missense_variant
BG_41408	*NOTCH1*	NM_017617.3:c.3394C>T	NP_060087.3:p.Arg1132Cys	20.6	194	40	missense_variant
BG_42228	*NOTCH1*	NM_017617.3:c.4778T>C	NP_060087.3:p.Leu1593Pro	48.7	189	92	missense_variant
BG_4255	*NOTCH1*	NM_017617.3:c.4776_4777insAGAACC	NP_060087.3:p.Phe1592_Leu1593insArgThr	19.6	168	33	inframe_insertion
BG_4255	*NOTCH1*	NM_017617.3:c.141-4A>G		5.2	155	8	splice_region_variant, intron_variant
BG_37265	*NRAS*	NM_002524.4:c.37G>C	NP_002515.1:p.Gly13Arg	19.3	1464	283	missense_variant
BG_41408	*NRAS*	NM_002524.4:c.35G>A	NP_002515.1:p.Gly12Asp	26.8	1131	303	missense_variant
BG_4379	*NRAS*	NM_002524.4:c.35G>A	NP_002515.1:p.Gly12Asp	43.4	2130	924	missense_variant
BG_5038	*PAX5*	NM_016734.2:c.780+5G>T		45.5	321	146	splice_region_variant, intron_variant
BG_21292	*PTEN*	NM_000314.4:c.493G>T	NP_000305.3:p.Gly165Ter	32.9	347	114	stop_gained, splice_region_variant
BG_21292	*PTEN*	NM_000314.4:c.736_737insAG	NP_000305.3:p.Pro246GlnfsTer11	34.3	1130	388	frameshift_variant
BG_37265	*SH2B3*	NM_005475.2:c.1345G>A	NP_005466.1:p.Glu449Lys	5.8	924	54	missense_variant
BG_39652	*SH2B3*	NM_005475.2:c.927-2delAGinsCT		5.4	112	6	splice_acceptor_variant
BG_4255	*SH2B3*	NM_005475.2:c.1038dupG	NP_005466.1:p.Leu347AlafsTer38	50.2	944	474	frameshift_variant
BG_39652	*TET2*	NM_001127208.2:c.1588delCAinsTG	NP_001120680.1:p.Gln530Trp	6.2	242	15	stop_gained
BG_41165	*TET2*	NM_001127208.2:c.5733delA	NP_001120680.1:p.Lys1911AsnfsTer39	35.7	493	176	frameshift_variant
BG_10442	*TP53*	NM_000546.5:c.684_685insGGGGTTTGACC	NP_000537.3:p.Cys229GlyfsTer3	5.9	236	14	stop_gained,frameshift_variant
BG_10442	*TP53*	NM_000546.5:c.651_654dupGGTG	NP_000537.3:p.Pro219GlyfsTer4	33.7	943	318	frameshift_variant
BG_11584	*TP53*	NM_000546.5:c.844C>G	NP_000537.3:p.Arg282Gly	4.6	1034	47	missense_variant
BG_41209	*TP53*	NM_000546.5:c.390_426delCAACAAGATGTTTTGCCAACTGGCCAAGACCTGCCCT	NP_000537.3:p.Asn131CysfsTer27	35.0	117	41	frameshift_variant

VAF: variant allele fraction; RD: read depth; ARD: alteration read depth.

## Supplementary Materials

The following are available online at https://www.mdpi.com/2072-6694/12/6/1505/s1, Table S1: List of genes included in the enhanced version of the capture-NGS panel (panel v2), Table S2: Clinical and biological characteristics of adult ALL patients evaluated for the validation of the capture-NGS panel v1, Table S3: Clinical and biological characteristics of adult ALL patients lacking an informative Ig/TCR molecular marker for MRD evaluation (MRD-unknown).
