# Supplementary material for: Capture-Based Next-Generation Sequencing Improves the Identification of Immunoglobulin/T-Cell Receptor Clonal Markers and Gene Mutations in Adult Acute Lymphoblastic Leukemia Patients Lacking Molecular Probes
Source: Cancers (Basel). 2020 Jun 9;12(6):1505. doi: 10.3390/cancers12061505 (PMC7352935; doi:10.3390/cancers12061505)
Supplement: Supplementary file 1 [file cancers-12-01505-s001.pdf]

Supplementary Files

Capture-based Next-Generation Sequencing Improves the Identification of Immunoglobulin/T-Cell Receptor Clonal Markers and Gene Mutations in Adult Acute Lymphoblastic Leukemia Patients Lacking Molecular Probes

Roberta Cavagna, Marie L. Guinea Montalvo, Manuela Tosi, Michela Paris, Chiara Pavoni, Tamara Intermesoli, Renato Bassan, Andrea Mosca, Alessandro Rambaldi and Orietta Spinelli

Table S1. List of genes included in the enhanced version of the capture-NGS panel (panel v2).

Gene Name	Chromosome
ALB	chr4
BRAF	chr7
CREBBP	chr16
EZH2	chr7
FBXW7	chr4
FLT3	chr13
IDH1	chr2
IDH2	chr15
IKZF1	chr7
IL7R	chr5
JAK1	chr1
JAK2	chr9
JAK3	chr19
KRAS	chr12
NOTCH1	chr9
NRAS	chr1
PAX5	chr9
PTEN	chr10
SH2B3	chr12
STIL	chr1
TAL1	chr1
TAL2	chr9
TET2	chr4
TP53	chr17
TYK2	chr19

Table S2. Clinical and biological characteristics of adult ALL patients evaluated for the validation of the capture-NGS panel v1, formerly studied for clonality assessment according to the conventional EuroMRD guidelines within the NILG-ALL 09/00 clinical trial. Patients have been selected for having at least two sensitive patient-specific probes (P1 and P2).

Patient ID	Cohort	Assay	Age at DX	Sex	ALL lineage	Blasts %	Karyotype	Probe 1 (P1)	P1 Assay Sensitivity	Probe 2 (P2)	P2 Assay Sensitivity
BG_371	R	v1	41	M	B	80	48,XY, +?8,+?13(9)/46,XY(12)	TRGV3*02-J1*02	1,00E-05	TRDV2*02-D3*01	1,00E-04
BG_4502	R	v1	28	M	T	100	46,XY del(6)(q21), t(9;10)	TRGV9*01-J1*02	1,00E-05	TRDV1*01-J1*01	1,00E-05
BG_5038	R	v1; v2	56	M	B	99	46,XY	TRDD2*01-DD3*01	1,00E-05	IGKV1-16*02-KDE	1,00E-05
BG_5418	R	v1	39	M	B	90	46,XY	IGHV3-7*02-J6*02	1,00E-05	TRGV9*01-J1*02	1,00E-05
BG_5452	R	v1	25	M	T	100	5q- (q31)	TRGV10*02-J1*02	1,00E-04	TRBD1-J2-3	1,00E-04
BG_9574	R	v1	46	M	T	80	46,X,inv(Y),del(6)(q21),t(10;14)(q24;q11)(12)/46idem,add(9)(p?)(4)	TRGV11*01-J2*01	1,00E-05	TRGV4*02-J1*02	1,00E-04
BG_9813	R	v1	16	F	T	90	46,XX. Molecular detection of *SIL-TAL1* fusion gene	TRDD2*01-J1*01	1,00E-05	TRGV9*01-J2*01	1,00E-04
BG_11360	R	v1	15	F	B	60	(46,XX), 1t(1;12), 1del(9)	TRDD2*01-D3*01	1,00E-04	TRDV2*02-D3*01	1,00E-04
BG_11720	R	v1	41	F	B	90	46,XX	TRGV9*01-J1*02	1,00E-05	IGHV6-1*01-J6*02	1,00E-05
BG_11806	R	v1	27	M	B	90	46,XY	TRGV5*01-J1*02	1,00E-04	IGHV1-2*02-J5*02	1,00E-05

R: Retrospective, v1: capture-based NGS panel targeting Ig/TCR loci, v2: capture-based NGS panel targeting both Ig/TCR loci and selected genes of interest, DX: Diagnosis.

Table S3. Clinical and biological characteristics of adult ALL patients lacking an informative Ig/TCR molecular marker for MRD evaluation (MRD-unknown).

Patient ID	Cohort	Assay	Age at DX	Sex	ALL lineage	Blasts %	Karyotype
BG_855	R	v1	63	F	B	90	46,XX
BG_1125	R	v1	43	M	B	90	Not Done
BG_2097	R	v1	62	M	B	60	47,XY,inv dup(iq)(ipter) 1q22;1q22cen;1q23)1qter),dup(2p)(2pter)2p13;2p13)qter),t(8;14)(8q24;14q32),del(13)(13pter)13q13;13q22)13qter),+mar,46,XY
BG_2481	R	v1	32	M	T	94	Absence of Metaphases
BG_3895	R	v1	20	M	T	47	46,XY
BG_4005§	R	v1	18	M	T	Na	46,XY
BG_4255	R	v2	36	M	T	80	46,XY
BG_4254	R	v1	63	F	B	90	63-65,XX(7)/46,XX(2)
BG_4379	R	v2	50	M	T	95	46,XY (10)
BG_5702	R	v1	62	F	B	85	Hyperdiploid (63 chr)
BG_6037	R	v1	33	F	T	95	Absence of Metaphases
BG_6490	R	v1	63	M	B	95	Triploidy in 5 Metaphases/15 normal Metaphases
BG_8646	R	v1	59	M	T	90	46,XY,del(5)(q22q35),del(12)(p11p13),del(17)(p11)(18); 46, XY (2)
BG_8345	R	v1	33	F	B	64	46,XX
BG_9445	R	v1	45	M	B	80	36,XY,-2,-3,-4,-7,-8,-9,-d,-16,-17,-20
BG_10112	R	v1	63	M	B	93	46,XY, t(4;11)(q21;q23)
BG_10487	R	v1	20	M	B	56	Not Evaluable
BG_10640	R	v1	62	F	B	83	Absence of Metaphases
BG_11053	R	v1	25	F	B	90	46,XX(18). Molecular detection of *KMT2A-AFF1* transcript from t(4;11)
BG_11269	R	v2	33	M	T	>50	46,XY
BG_11345	R	v1	63	M	T	90	48,XY+10+21/47XY, 10/47,XY+21/46XY
BG_11584	R	v2	38	F	B	100	Absence of Metaphases. Molecular detection of *KMT2A-AFF1* transcript from t(4;11)
BG_12438	R	v1	26	M	T	>50	46,XY
BG_37265	P	v2	52	M	T-LL	5-10	46,XY (15). Poor quality metaphases
BG_39541*	P	v2	28	M	T	5	Na
BG_39652*	P	v2	35	M	T-LL	30	Na
BG_40129*	P	v2	43	M	T	96	Na
BG_41165*	P	v2	63	M	T	71	Na
BG_41182*	P	v2	50	M	B	95	Na
BG_41209*	P	v2	20	M	T	63	Na
BG_41408*	P	v2	36	M	T	70	Na
BG_41733*	P	v2	21	M	T	70	Na
BG_41985*	P	v2	21	M	B	95	Na
BG_42228*	P	v2	44	M	T	83	Na
BG_42309	P	v2	75	F	B	70	46,XX (4). Low number of metaphases
BG_10442¶	G	v2	44	M	T	40	54,XY,+5?,+7,+8,+8,+15?,+21?,+21,+22(6)/46,xy(1)
BG_21292*¶	G	v2	43	M	T	99	Na. Molecular detection of *SIL-TAL1* fusion gene

§For patient BG_4005 the blasts percentage in the diagnostic sample was Not available (Na). *For patients indicated with "*", the karyotype evaluation was Not available (Na), ¶For patients BG_10442 and BG_21292 the Ig/TCR clonal markers assessment has been performed at diagnosis by standard procedure and not included in our study. Samples deriving from those patients have been used for the validation of the capture-based NGS panel v2 targeting both Ig/TCR loci and genes of interest (G). R: Retrospective, P: Prospective, G: Gene panel validation, v1: capture-based NGS panel targeting Ig/TCR loci, v2: capture-based NGS panel targeting both Ig/TCR loci and selected genes of interest, DX: Diagnosis.

© 2020 by the authors. Licensee MDPI, Basel, Switzerland. This article is an open access article distributed under the terms and conditions of the Creative Commons Attribution (CC BY) license (http://creativecommons.org/licenses/by/4.0/).
